# Rheumatic Aortic Regurgitation in a Patient with Large Congenital Fenestrations in All Three Leaflets

**DOI:** 10.5681/jcvtr.2014.012

**Published:** 2014-03-21

**Authors:** Ahmadreza Jodati, Babak Kazemi, Naser Safaei

**Affiliations:** Cardiovascular Research Center, Tabriz University of Medical Sciences, Tabriz, Iran

**Keywords:** Aortic Valve, Valve Fenestration, Aortic Regurgitation

## Abstract

Fenestrations of the aortic valve rarely produce significant valvular regurgitation. These are typically described as incidental findings with little clinical significance because they generally lie above their closing edges. Rarely however, when unusually large or multiple, they can lead to massive aortic regurgitation (AR), mostly in patients with chronic hypertension and/or aortic annular dilation. We operated a 52 year old normotensive male with chronic rheumatic AR and found large fenestrations in all three aortic cusps, hardly ever reported in rheumatic valvular involvement in the literature.

## Introduction

Fenestrations of the aortic valve rarely produce significant valvular regurgitation. These are typically described as incidental findings with little clinical significance because they generally lie above their closing edges.^1,2^ Rarely however, when unusually large or multiple, they can lead to massive aortic regurgitation (AR), mostly in patients with chronic hypertension and/or aortic annular dilation.^2-4^

## Case report

A 52 year old male originally from Azerbaijan was referred to us for evaluation of progressive effort angina and dyspnea. Cardiac auscultation revealed a normal S1 and decreased S2, a grade 3/6 diastolic blowing murmur along the left sternal border and across the precordium, and a grade 2/6 holo-systolic murmur at the apex. At echocardiography, the aortic valve was thick and calcified, suggesting rheumatic disease, with severe regurgitation. There was no annular dilation (annular and sinotubular dimensions were 27 and 36 mm, respectively). The mitral valve was thick and moderately regurgitant, without prolapse. The left ventricle showed moderate dilation, mild wall thickening, a global ejection fraction of 47% and an effective ejection fraction of 30%, and features suggestive of an elevated end-diastolic pressure. Right ventricular size and function were normal. No valvular vegetations were detected, and blood cultures were consistently negative. Due to the severity of his AR and presence of left ventricular dysfunction, surgical intervention was recommended. Surgery revealed a malformed aortic valve with 3 equal-sized cusps, each of which had a single large central fenestration (Figure 1-A). The valve was removed and replaced with a 27-mm St. Jude aortic prosthesis, and the patient recovered without incident. Microscopic study showed degenerative changes with fibrosis, sclerosis, and calcification suggestive of rheumatic involvement (Figure 1-B & C).

Figure 1
Aortic valve leaflets after operation with large fenestrations present in each leaflet. (11x6 mm in both right and left leaflets and 7x3 mm in the noncoronary leaflet) (A). Degenerative changes with fibrosis and sclerosis (B) and calcification (C) are present in microscopic evaluation of the leaflets (Eosin-haematoxylin stain; ×100).

**A:**
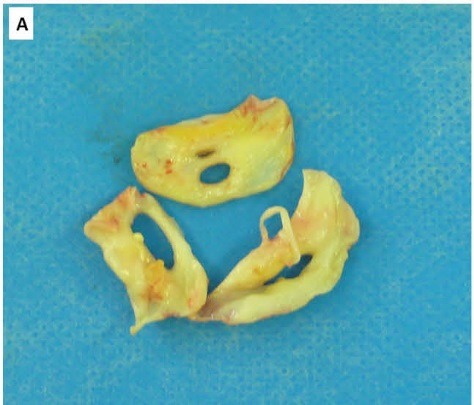

**B:**
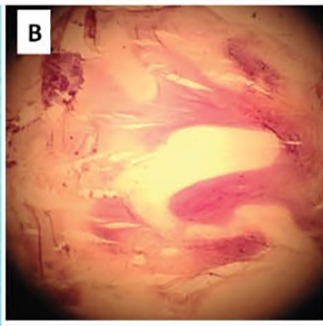

**C:**
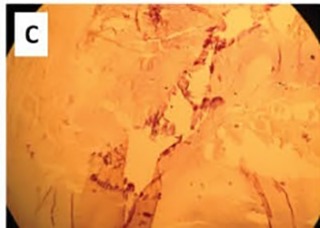

## Discussion

Between the closing and free edges of each aortic cusp are two crescent-shaped lunular areas, one on each side of the nodule of Arantius. During valve closure, coaptation occurs across the entire lunular surface, thereby implementing a competent seal. With increasing age, fenestrations often form within the lunular regions, near the commissures, and may be single or multiple.^1^ Because of their location above the line of closure, most age-related fenestrations do not result in regurgitation. However, chronic valvular incompetence can occur if a fenestration is unusually large and extends below the closure line or if the valve annulus dilates and causes cusp stretching and prolapse, which in that case, proper coaptation of the lunular areas of two opposing cusps will be lost.^3,5,6^

Hope in year 1842 and Rokitansky in 1852 were first to describe aortic leaflet fenestration.^7,8^ Foxe conducted the first systematic study on fenestrations of the semilunar valves of the human heart in 300 consecutive autopsies and found that there were one or more fenestrations in 82% of all cases.^1^ Most of these fenestration were small and clinically insignificant.He also concluded that the frequency of fenestrations increases with age from the fetus to the fourth decade, and after this there is a slight decrease, possibly due to the increasing frequency of sclerotic changes which obliterate small defects.^1^

The presence of large fenestrations in all aortic valve leaflets leading to severe chronic AR has been rarely reported.^3,6,9^ There is one case report on the presence of large fenestrations in all four leaflets of a quadricuspid aortic valve leading to AR.^10^In almost all cases the histologic findings of the affected aortic leaflets have suggested myxomatous degeneration.^3,6,9-11^

In a case series of 6 patients with AR and fenestrated leaflets reported by Akiyama et al.^10^ who were candidate for surgery, none of them showed systemic connective tissue disease or annuloaortic ectasia, history of infective endocarditis or chest trauma. At surgery, fenestrations were located within every cusp at all 3 commissures in 5 patients, and the other case had fenestrations within only 2 cusps. The histopathological findings of the excised aortic valves showed myxomatous degeneration in all 6 cases.

Our case was unique in a way that it occurred in a patient with rheumatic involvement of the aortic valve, which has never been reported. The main reason for the rarity of this finding may be, as previously stated, the obliteration of small fenestrations and preventing them from enlargement in sclerotic and thickened leaflets due to rheumatic involvement. Although large aortic valve fenestrations can produce chronic AR like our patient, acute rupture of the fenestrated fibrous cord, either spontaneously^4,12,13^ or because of infection,^14^ can also lead to massive AR resulting in acute left heart failure.

As there is a steady decrease in the prevalence of rheumatic heart disease, even in most developing countries around the world, and the mean age of the population are in the rise, we must expect an increased incidence of AR caused by aortic fenestrations, especially in those with chronic systemic hypertension. We should be aware of this abnormal entity in any patient with chronic AR or sudden deterioration of AR, if there is not any other obvious cause. Aortic valve anatomy can be clearly defined by transthoracic echocardiography in most patients but for detecting leaflet fenestrations usually transesophageal echocardiography is needed. We did not suspect leaflet fenestrations in our patient at transthoracic echocardiography because of typical rheumatic thickening of the leaflets and did not perform transesophageal echocardiography before operation. Suggestive findings for AR caused by a fenestrated aortic valve are an enlarged aortic annulus with a diameter greater than 30 mm, a prolapsing right coronary cusp with or without eccentric regurgitation, and an abnormal fibrous band attached to the prolapsing cusp or fibrous band between the aortic cusp and the aortic wall at the commissure.^10^ Surgical repair is usually possible in the presence of one or two fenestrations without concomitant aortic root dilation, but when present in all three leaflets or unusually large aortic valve replacement seems to be the better strategy.^10,15^

Our center is a tertiary referral heart center for the north-west of Iran with more than 2000 operations performed on the aortic valve in the last 45 years and we haven’t encountered any case with rheumatic involvement and large fenestrations in all three leaflets leading to chronic AR.

## Ethical issues

The study was approved by the ethics committee of the University.

## Competing interests

The authors had no competing interests to declare in relation to this article.
